# Characteristics and Prognostic Factors of Non-HIV Immunocompromised Patients With Pneumocystis Pneumonia Diagnosed by Metagenomics Next-Generation Sequencing

**DOI:** 10.3389/fmed.2022.812698

**Published:** 2022-03-03

**Authors:** Jiali Duan, Jing Gao, Qiuhong Liu, Mengfei Sun, Yang Liu, Yingshuai Tan, Lihua Xing

**Affiliations:** Department of Pulmonary and Critical Care Medicine, The First Affiliated Hospital of Zhengzhou University, Zhengzhou, China

**Keywords:** immunocompromised, *Pneumocystis jirovecii*, pneumonia, non-HIV, mNGS

## Abstract

**Objective:**

The aim of this study was to evaluate the potential of metagenomic next-generation sequencing (mNGS) for the diagnosis of pneumocystis pneumonia (PCP) in patients with non-human immunodeficiency virus-infection and to discuss the clinical characteristics and identify prognostic factors associated with patients with non-HIV PCP.

**Methods:**

Forty-six patients with PCP who were admitted in respiratory intensive care unit (ICU) between May 2018 and May 2020 were retrospectively reviewed. The subjects were divided into survivor and non-survivor groups according to the patients' outcome. Conventional methods and mNGS for detecting *Pneumocystis jirovecii (P. jirovecii)* were analyzed. The patients' demographics, comorbidities, laboratory parameters, and treatments were compared and evaluated in both groups to identify risk factors for mortality by using univariate and multivariate logistic regression.

**Results:**

Metagenomic next-generation sequencing (mNGS) showed a satisfying diagnostic performance of 100% positive of detecting *P. jirovecii* from bronchoalveolar lavage (BAL) specimens in forty-six patients with non-HIV PCP, compared to only 15.2% for Gomori Methenamine silver (GMS) staining and 84.8% for Serum 1,3-beta-D-glucan (BDG). Among them, the mean age was 46.4-year-old (range 18–79-year-old) and mortality rate was 43.5%. The dominant underlying conditions were connective tissue diseases (34.8%), autoimmune kidney diseases (30.4%), followed by hematologic malignancies (10.9%), and solid organ transplantation (6.5%). A total of 38 cases (82.6%) received glucocorticoid and 19 cases (41.3%) used immunosuppressant within 3 months before diagnosed PCP. Multiple infections were very common, over two thirds' cases had mixed infections. Compared with survivors, non-survivors had a higher acute physiology and chronic health evaluation II (APACHE II) score (14.4 ± 4.8 vs. 10 ± 3.4), Procalcitonin (PCT) [ng/ml: 0.737 (0.122–1.6) vs. 0.23 (0.095–0.35)], lactic dehydrogenase (LDH) [U/L: 1372 (825.5–2150) vs. 739 (490.5–956)], and neutrophil-lymphocyte ratio (NLR) [21.6 (15.67–38.2) vs. 11.75 (5.1–15.52)], but had a lower PaO_2_/FiO_2_ ratio (mmHg:108.8 ± 42.4 vs. 150.5 ± 47.5), lymphocytes [×10^9^/L: 0.33 (0.135–0.615) vs. 0.69 (0.325–1.07)] and CD4+ T cells [cell/μl: 112 (53.5–264) vs. 255 (145–303.5)], all *P* < 0.05. Furthermore, we found non-survivors' PaO_2_/FiO_2_ ratio of day 3 and day 7 had not improved when compared with that of day one, and platelet level and NLR became worse. Multivariate analysis showed that other pathogens' co-infection (OR = 9.011, 95% CI was 1.052–77.161, *P* = 0.045) and NLR (OR = 1.283, 95% CI was 1.046–1.547, *P* = 0.017) were the independent risk factors of poor prognosis.

**Conclusion:**

mNGS is a very sensitive diagnostic tool for identifying *P. jirovecii* in patients who are non-HIV immunocompromised. PCP in patients who are non-HIV infected is associated with a high rate of multiple infections and severe condition. Mixed infection and elevation of NLR were the independent risk factors of poor prognosis.

## Introduction

Pneumocystis pneumonia (PCP) is an opportunistic pulmonary fungal infection caused by *Pneumocystis jirovecii* (*P. jirovecii*) in immunocompromised populations. And it is very common among patients with HIV/AIDS. Especially, patients with HIV/AIDS with a low CD4 count (CD4 counts <200/μl) are at the highest risk of PCP (1, 2). With the widespread use of antiretroviral and PCP prophylaxis therapy, the incidence and mortality of PCP in patients with HIV/AIDS has gradually declined and it is around 10–20% nowadays (3, 4). However, the emerging number of conditions associated with immunosuppression has led to its continuing appearance in non-HIV patient population. The incidence of PCP is gradually increasing among patients who are non-HIV immunocompromised such as those receiving chronic corticosteroid therapies, with hematological or solid malignancies, transplant recipients and those who receive immunomodulatory or biological therapy (5, 6). Moreover, in the non-HIV-infected population, PCP is often associated with a higher rate of progressing to respiratory failure which occurs within a short time and causes a mortality rate of 30–60% (7). The growing incidence and high mortality of pneumocystis infection in the non-HIV patients suggest the need of more attention, including earlier diagnosis and treatment.

Despite the growing prevalence of PCP in immunocompromised individuals, establishing a microbiological diagnosis remains a challenge in this vulnerable population. Because the Inability to culture pneumocystis and non-specific clinical manifestations, together with relatively lower burden of organisms in HIV-uninfected patients (4, 8), conventional microbiological methods usually have an inadequate performance of finding *P. jirovecii*, and it may present false negative results of conventional tests (9). Metagenomics next-generation sequencing (mNGS) is a molecular technology of nucleic acid sequencing with high-throughput capacity and unbiased pathogen detection in a single assay, which has been considered a promising microbial identification technology in infectious diseases (10). Recently, several studies have shown its advantage in detecting a wide range of pathogens from different clinical specimens (11–13). However, the study about the utility of mNGS of bronchoalveolar lavage (BAL) fluid specimens for the diagnosis of PCP in non-HIV patients remains rare.

In the present study, we describe the use of mNGS of BAL fluids for detecting *P. jirovecii* and discuss the characteristics and risk factors of the outcome of non-HIV immunocompromised patients with PCP.

## Materials and Methods

### Study Participants

We retrospectively collected a total of 46 immunocompromised patients diagnosed with PCP by mNGS test of BAL in our respiratory intensive care unit (ICU) from May 2018 to May 2020. This study was approved by the ethical committee of the first affiliated hospital of Zhengzhou University (The ethics approval number was 2020-KY-521), and informed consents were signed by patients or surrogates. Patients who met the following criteria were diagnosed with PCP: (1) Clinical symptoms (cough, fever, or shortness of breath) and laboratory parameters [1,3-beta-D-glucan (BDG) test and lactic dehydrogenase (LDH)] relevant to PCP; (2) Imaging findings compatible with PCP (present bilateral interstitial, ground-glass opacity and alveolar infiltrates in perihilar areas); (3) Identifying the genetic sequences of *P. jirovecii* by mNGS of BAL specimens. All patients enrolled were defined as HIV-negative, with one or more of the following immunosuppressive host conditions: receiving corticosteroid therapy within 3 months; active malignancy and/or receiving cancer chemotherapy; solid organ transplantation; hematological malignancy; receiving biologic immune modulators and/or immunosuppressive therapy. Patients with the following conditions were excluded: (1) Age < 18 years old; (2) HIV infection; (3) Length of stay in ICU < 24 h; (4) Incomplete medical record.

### Microbiological Investigations

All patients had bronchoscopy performed, and BAL specimens were obtained. Each BAL specimen was divided into aliquots for both conventional microbiological test and mNGS test. Some specimens were sent to the Vision Medical Co., Ltd. (China) for the mNGS analysis, and the processes of nucleic acid extraction, library construction, and high-throughput sequencing were performed, in turn. Finally, bioinformatics analysis and pathogen data interpretation were performed, all of which were already noted in detail in previous studies (14, 15).

At the same time, conventional tests were also conducted for microbiological analysis, including bacterial/fungal smear and culture, acid-fast staining, *P. jirovecii* smear [Gomori Methenamine silver (GMS) staining], and real-time PCR including *cytomegalovirus* (CMV), *influenza virus*. Real-time PCR was performed with a commercial CMV assay using MagNA Pure LC instrument (Roche Molecular Biochemicals, Indianapolis, IN). Positive control and negative control were included in all assays. The lower limit of detection of the assay was estimated to be <50 copies/ml (as per the instruction of the manufacturer). DNA integrity of the samples was confirmed by the presence of internal control DNA. The mixed infection was defined as the isolation of more than one pathogenic species. Furthermore, there was a consensus of clinically significant pathogens reached by physicians based on the comprehensive analysis of conventional and mNGS results, clinical features, and laboratory findings.

### Clinical Data Collection

Clinical parameters of each patient were acquired through review of electronic medical records. We recorded patient data regarding demographics, underlying diseases, use of immunosuppressant, laboratory test results, acute physiology and chronic health evaluation II (APACHE II) score, the time of ICU stay, and the outcome of the patient.

### Statistical Analysis

For continuous variables, normally distributed variables were reported as the mean and standard deviation and as the median and interquartile range (IQR) if they had a skewed distribution. Categorical variables were expressed as frequencies and percentages. Continuous variables were compared using the *t*-test or Mann-Whitney U test where suited. Categorical variables were compared using the chi-squared or Fisher's test, and repeated-measures analysis of variance was used when comparing differences between continuous variables over time. Logistic regression models were used to identify the risk factors of poor outcome. Covariates with a *P* < 0.05 in the univariate analysis were included in the final model of multivariable logistic regression. All statistical analyses were performed using the SPSS software version 23.0 (IBM, Armonk, NY, USA). *P* < 0.05 were considered significant, and all tests were 2-tailed.

## Results

### Characteristics of Study Subjects and Detection of *P. jirovecii* in BAL Specimens by mNGS

Forty-six patients were enrolled during the study period. Among them, the mean age was 46.4 (18–79) years old and 22 (47.8%) of the patients were male. The mean age and gender compositions of survival and non-survival groups were similar. The underlying diseases of PCP patients were as follows: connect tissue diseases (34.8%), autoimmune kidney disease (30.4%), hematological malignancies (10.9%), and kidney transplant recipients (6.5%). As expected, PCP patients had various immunosuppressive conditions: Within 3 months prior the PCP diagnosis, 38 patients (82.6%) received steroids for inflammatory and/or autoimmune diseases, 19 cases (41.3%) took more than one immunomodulatory drug. However, none of them received PCP prophylaxis therapy (As shown in Table 1).

**Table 1:** Underlying and co-infection conditions of non-HIV immunocompromised pneumocystis pneumonia (PCP) patients.

**Item**	**Total**	**Survivors**	**Non-survivors**
	**(*n* = 46, %)**	**(*n* = 26, %)**	**(*n* = 20, %)**
**Underline conditions**			
Connect tissue disease	16 (34.8)	10 (38.5)	6 (30)
Chronic kidney disease	14 (30.4)	7 (26.9)	7 (35)
Hematological malignancies	5 (10.9)	3 (11.5)	2 (10)
Solid organ transplantation	3 (6.5)	2 (7.7)	1 (5)
Solid tumors	2 (4.3)	1 (3.8)	1 (5)
Others	6 (13)	5 (19.2)	1 (5)
Use of corticosteroids	38 (82.6)	20 (76.9)	18 (90)
Use of immunosuppressive medications	19 (41.3)	10 (38.5)	9 (45)
**Mixed infections**			
No	15 (32.6)	14 (53.8)	1 (5)
Yes	31 (67.4)	12 (46.2)	19 (95)
Virus co-infection	23 (50)	11 (42.3)	12 (60)
*Cytomegalovirus*	19 (41.3)	9 (34.6)	10 (50)
Other viruses (*EB virus, Human adenovirus 7, and Human alpha herpesvirus 1*)	4 (8.7)	2 (7.7)	2 (10)
Bacteria co-infection	13 (28.3)	6 (23.1)	7 (35)
*Klebsiella pneumoniae*	4 (8.7)	2 (7.7)	2 (10)
*Peudomonas aeruginosa*	5 (10.9)	3 (11.5)	2 (10)
*Acinetobacter baumannii*	3 (6.5)	1 (3.8)	2 (10)
*Staphylococcus aureus*	1 (2.2)	0 (0)	1 (5)
Fungus co-infection	11 (23.9)	4 (15.4)	7 (35)
*Candida albicans*	6 (13)	2 (7.7)	4 (20)
*Aspergillus fumigatus*	3 (6.5)	1 (3.8)	2 (10)
Other fungi *(Candida tropicalis, Aspergillus flavus)*	2 (4.3)	1 (3.8)	1 (5)

Metagenomics next-generation sequencing (mNGS) and GMS staining of BAL specimens were performed in each patient. The mNGS test of forty-six patients' specimens all found *P. jirovecii* (sequence reads 12–214,898), and the result of GMS staining of BAL specimens showed that *P. jirovecii* was identified only in 7 cases (15.2%). Additionally, sputum specimens were obtained from 18 patients (39.1%); however, all showed negative results of *P. jirovecii*. BDG was widely used as a serologic biomarker of PCP. In this study, Serum BDG testing showed that 84.8% of patients had a positive result (Serum BDG > 100 ng/L).

### Treatments and Outcome

All patients were treated with trimethoprim-sulfamethoxazole or combined with caspofungin. Meanwhile, 42 patients (93.5%) received adjuvant corticosteroid therapy. As for oxygen support, 21 patients needed invasive mechanical ventilation, the remaining patients were oxygenated by non-invasive mechanical ventilation and high-flow nasal oxygen treatment. The non-survival group had a higher rate of receiving invasive mechanical ventilation when compared with the survival group (70% vs. 26.9%, *P* = 0.004).

Multi-infections were very common among PCP patients, and about 2/3 of patients had mixed infections. Among them, the most commonly detected co-infection pathogen was CMV. A total of 19 cases were co-infected with CMV, but there was no significant difference on the incidence between the two groups. Although patients with multi-infections were prescribed with relevant anti-bacterial or anti-fungal, antiviral agents, patients with multiple infections still had higher risk of poor outcomes than those without. Non-survivors had a significantly higher rate of mixed infection (95% vs. 46.2%, *P* = 0.001), which was also shown to be an independent risk factor of death in multivariate logistic analysis. The hospital mortality rate of the patients was 43.5%. The length of stay in the ICU of non-survivors was shorter than those of the survivors, but no statistical significance was observed (As shown in Table 2).

**Table 2:** Demographic and clinical characteristics of non-HIV PCP patients.

**Variable**	**Total (***n*** = 46)**	**Survivors (***n*** = 26)**	**Non-survivors (***n*** = 20)**	***P*** **-value**
Male, *n* (%)	22 (47.8)	12 (46.2)	10 (50)	0.796
Age, year	46.4 ± 15	45.8 ± 14.9	47.2 ± 15.5	0.78
APACHE II score	12.2 ± 3.9	10 ± 3.4	14.4 ± 4.8	0.042
PaO_2_/FiO_2_ ratio, mmHg	133.6 ± 49.4	150.5 ± 47.5	108.8 ± 42.4	0.006
Corticosteroid use, *n* (%)	38 (82.6)	20 (76.9)	18 (90)	0.246
Immunomodulatory medication use, *n* (%)	19 (41.3)	10 (38.5)	9 (45)	0.655
**Laboratory tests**
White blood cells, × 10^9^/L	9.1 ± 4.2	8.4 ± 4.3	10.2 ± 3.8	0.185
Neutrophils, × 10^9^/L	7.85 (5.6–11.07)	6.9 (4.27–11)	9.05 (7.05–12.55)	0.075
Lymphocytes, × 10^9^/L	0.55 (0.25–0.82)	0.6 (0.325–1.07)	0.33 (0.135–0.615)	0.021
NLR	14.6 (7.85–21.65)	11.7 (5.1–15.52)	21.6 (15.67–38.2)	<0.001
Hemoglobin, g/L	109.6 ± 21.1	109.5 ± 22.5	109.8 ± 19.5	0.96
Platelet, × 10^9^/L	163.7 ± 71.2	176.6 ± 67.7	144.8 ± 73.9	0.158
C-reactive protein, mg/L	63.4 (30.5–117.4)	58.2 (30–83.7	112.7 (35.5–153.2)	0.056
Procalcitonin, ng/ml	0.238 (0.099–0.64)	0.2 (0.095–0.35)	0.737 (0.122–1.6)	0.035
Lactate dehydrogenase, U/L	891 (562–1701.5)	739 (490.5–956)	1,372 (825.5–2150)	0.003
Serum albumin, g/L	29.4 ± 4.3	29.9 ± 4.1	28.7 ± 4.6	0.39
Serum BDG, >100 ng/L (%)	39 (84.8)	21 (80.8)	18 (90)	0.388
IL-6, pg/ml	37.78 (13.1–166.3)	34.1 (13.1–166.3)	41.1 (13.25–190.33)	0.663
IL-10, pg/ml	12.5 (6.97–22.12)	12.77 (6.88–18.23)	12.42 (6.79–39.73)	0.758
TNF-α, pg/ml	2.44 (1.47–3.51)	2.40 (1.35–3.22)	2.47 (1.77–4.47)	0.377
CD4+T lymphocytes,/μL	179.5 (101.5–299.8)	255 (145–303.5)	112 (53.5–264)	0.046
CD8+T lymphocytes,/μL	179 (104–287)	200 (104–290)	124 (92–286)	0.311
CD4+T/CD8+ ratio	1.03 (0.69–1.5)	1.36 (0.78–1.73)	0.8 (0.63–1.05)	0.006
Multiple infections, *n* (%)	31 (67.4)	12 (46.2)	19 (95)	0.001
**Treatment**
IMV, *n* (%)	21 (45.7)	7 (26.9)	14 (70)	0.004
Glucocorticoid, *n* (%)	42 (91.3)	23 (88.5)	19 (95)	0.435
ICU time, d	10.5 (8.5–15.3)	12 (9.5–16)	10 (6.5–12.5)	0.068

*APACHE II, acute physiology and chronic health evaluation II; NLR, neutrophil-lymphocyte ratio; BDG, 1,3-beta-D-glucan; IMV, invasive mechanical ventilation; ICU, Intensive care unit*.

### Prognostic Factors by Univariate Analysis

As shown in Table 2, compared with survivals, non-survivals appeared to have higher white blood cell count, neutrophil count and serum C-reactive protein, but no significance was observed between the two groups. All PCP patients showed lymphopenia with a median lymphocyte count of 0.55 × 10^9^/L in peripheral blood and had significantly high LDH levels. Median serum levels of LDH (739 vs. 1,372 U/L) and neutrophil-to-lymphocyte ratio (NLR) (11.75 vs. 21.6) remarkably increased in both cohorts. Compared with survivors, non-survivors had a significantly higher APACHE II score within 24 h while admitted in the ICU, higher NLR ratio, PCT and LDH level, but had a lower PaO_2_/FiO_2_ ratio, lower lymphocytes, CD4+T cells and CD4+/CD8+ ratio; all *P* < 0.05. There was no significant difference in comparison of the cytokine' levels (IL-6, IL-10, and TNF-α) between the two groups.

### Multivariate Analysis

Covariates with a *P* < 0.05 in the univariate analysis were included in the final model of multivariable logistic regression, and multivariate analysis showed that multiple infection and NLR were independent risk factors for non-HIV PCP patients that resulted in death (OR 1.283; 95% CI 1.046–1.574; OR 9.011; 95% CI 1.052–77.161; respectively) (Shown in Table 3).

**Table 3:** Univariate and multivariate analysis of risk factors for poor outcome.

**Variable**	**Univariate analysis**			**Multivariate analysis**		
	**OR**	**95% CI**	***P*** **-value**	**OR**	**95% CI**	***P*** **-value**
PaO_2_/FiO_2_, mmHg	0.979	0.963–0.995	0.012	0.985	0.960–1.010	0.227
APACHE II score	1.156	1.000–1.336	0.049	0.939	0.702–1.225	0.669
Lymphocytes, × 10^9^/L	0.13	0.02–0.857	0.034	0.436	0.031–6.113	0.538
NLR	1.227	1.067–1.412	0.004	1.283	1.046–1.574	0.017
CRP, mg/L	1.014	1001–1.026	0.03	1.008	0.985–1.031	0.492
LDH, U/L	1.001	1.000–1.002	0.03	1.001	1.000–1.003	0.076
CD4+T/CD8+T ratio	0.148	0.029–0.759	0.022	0.331	0.009–1.196	0.123
co-infection with other pathogens	8.545	1.791–50.882	0.008	9.011	1.052–77.161	0.045

*APACHE II, acute physiology and chronic health evaluation II; NLR, Neutrophil-to-lymphocyte ratio; CRP, C-reactive protein; LDH, lactic dehydrogenase*.

### Dynamic Changes of Laboratory Parameters Between Survival and Non-survival PCP Patients

**Figure 1:**
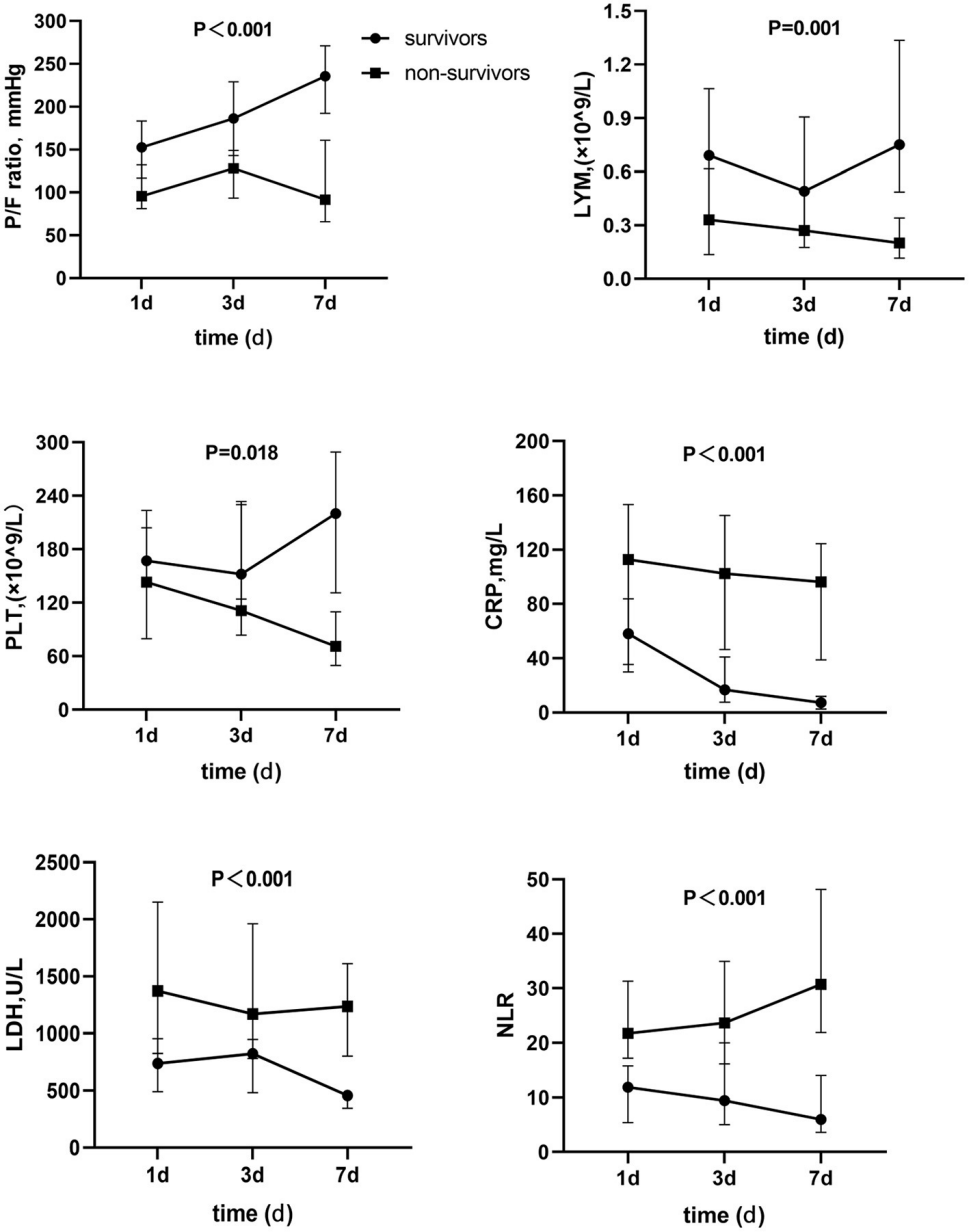
Dynamic change of parameters between survivors and non-survivors. P/F ratio, PaO_2_/PiO_2_ ratio; LYM, Lymphocyte; PLT, Platelet; CRP, C reactive protein; LDH, Lactate dehydrogenase; NLR, Neutrophil-to-lymphocyte ratio.

Figure 1 showed the survivor and death groups' comparison on the dynamic changes of several laboratory parameters on day 1, day 3, and day 7 in ICU. Apparently, PaO_2_/FiO_2_ ratio of survival patients was improved gradually. PaO_2_/FiO_2_ ratios on day 3 and 7 were obviously higher than day 1. However, non-survivors hadn't gained PaO_2_/FiO_2_ ratio's elevation, and comparison of the tendency of PaO_2_/FiO_2_ ratio between the two groups also showed significant difference. In addition, the lymphocyte level had increased gradually and tended to be normal. C-reactive protein (CRP) and LDH levels decreased among survivors, all of which presented converse change in non-survivors. Furthermore, non-survivors had a significant decrement of platelets and an obvious rise of NLR ratio, and the repeated-measures analysis of these variance showed significant difference; all *P* < 0.05.

## Discussion

With a growing incidence among immunocompromised populations, PCP became a more common opportunistic infection which can be life-threatening. In this retrospective study of 46 immunocompromised patients in ICU settings, all patients were diagnosed with PCP infection based on mNGS of BAL fluids, and none of the subjects had received any PCP prophylaxis. Meanwhile, mixed infections were very common among PCP patients. The hospital mortality rate was 43.5%. Multivariate analysis showed that mixed infection and NLR were independent risk factors of poor prognosis.

Conventional method *via* direct staining and microscopy for the detection of *P. jirovecii* lacks sensitivity. Furthermore, the organism cannot be cultured in a laboratory (7, 16). In the present study, mNGS which is a burgeoning microbial detection method confirmed the diagnosis of PCP among these patients. Genetic sequences of *P. jirovecii* were founded by mNGS from the BAL specimen of each patient while GMS staining only showed 15.2% positive results. Compared with the inadequate sensitivity of conventional methods (9), we found that mNGS had an excellent capability for the diagnosis of PCP and also had advantages in identifying co-pathogens in PCP patients with mixed infections. Again, data from several recent studies supported its advantages in detecting opportunistic pathogens and mixed infections, especially in detecting uncultivable pathogens, such as *P. jirovecii* (17–19). More importantly, in a retrospective study of evaluating the utility of mNGS for the diagnosis of PCP, their research data revealed that the sensitivity of mNGS was 100% in non-HIV infected PCP patients, which was dramatically higher than GMS staining and serum BDG (20).

Previous studies showed that PaO_2_/FiO_2_ ratio and invasive mechanical ventilation were the influence factors of PCP prognosis (21, 22). Consistent with these studies, we also found that non-survivors had a higher rate of invasive mechanical ventilation and lower PaO_2_/FiO_2_ ratio level. Moreover, non-survivors had almost gained no PaO_2_/FiO_2_ ratio's elevation during the first 7 days in ICU while PaO_2_/FiO_2_ ratio of survivors was improved gradually and was obviously higher than those of the non-survivors.

The elevation of LDH was shown to be correlated with PCP patients' poor outcome (23) in the study of Schimdt et al. Meanwhile, Yoshida et al. found that hypoproteinemia also affects prognosis among PCP patients with inflammatory bowel disease (24). By comparing the dynamic change of laboratory parameters (PaO_2_/FiO_2_ ratio, LDH and NLR, and so on) on day 1, 3, and 7 after admission in the ICU, our study demonstrated a significant different trend between survivors and non-survivors. Briefly, the non-survival group showed an increase in NLR and a decrement in platelets gradually, but gained no improvement in PaO_2_/FiO_2_ ratio. At the same time, the increase of CRP and LDH, which would indicate the ineffective response to treatment and the high risk of poor prognosis, was also significantly higher than that of the survivors' group. Similarly, the research on risk factors of short-term outcome in PCP patients with HIV infection showed that over 90% of patients had an abnormal test of CRP, ESR, PaO_2_, LDH, and KL-6 (25). In the present study, several laboratory parameters of PCP patients with poor prognosis got worse over the first 7 days in the ICU. Therefore, identifying the sickest patients early and taking the appropriate measures in a timely manner would improve the patient's outcome.

Because of depressive immune status, together with lower lymphocytes and T lymphocyte subsets than normal level, except PCP infection, these patients also had high risks of multiple infections occurring. The research of Huang et al. (26) of PCP patients with acute respiratory failure showed that occurrence of sepsis shock, ventilation related pneumonia, and CMV infection in PCP patients were 36.6, 43.9, and 58.5%, respectively. The result of another study about non-HIV PCP patients with CMV co-infection (27) indicated that fungus co-infection was a risk factor of CMV happening on PCP patients. Our study still found that mixed infections were very common in over 2/3 of patients with PCP, and the occurrence of mixed infection was also associated with poor prognosis. Multiple infections therefore need intense attention among immunocompromised PCP patients. Multilogistic regression analysis also showed NLR was a predictor of poor outcome. The elevation of NLR may be correlated with mixed infection and progress of the disease. The parameters of neutrophil and lymphocyte can be gained easily from regular daily blood test, so NLR would be a convenient and effective indicator which can be used to assess the patients' condition and prognosticate an outcome dynamically. However, there still need to be more prospective research to confirm its accuracy.

There is a need to mention that none of the patients in this study had received prophylaxis with sulfamethoxazole-trimethoprim, which would contribute to an occurrence of PCP in these patients. Almost similarly, the prophylaxis rates were all <20% in several previous studies on patients with PCP and non-HIV infection (21, 23, 28). Importantly, PCP prevention therapy can significantly reduce the morbidity and mortality of patients with PCP and HIV or patients with PCP but without HIV infection (29). There were already several clinical practice guidelines issued which suggest that the kidney transplant recipients and hematological malignancy patients should conduct PCP prophylaxis (30, 31). It is important that physicians should realize that non-HIV immunocompromised patients are also at risk of PCP and that rapid diagnosis and early initiation of treatment can lead to better prognosis. More attention is still needed to be paid on PCP prophylaxis among those non-HIV-infection populations with immunosuppression.

Our study had several limitations. First, the subjects were recruited from a single medical center, and the number of study participants was relatively small. Second, this was a retrospective study, some biases (such as selective bias) may influence the accuracy of our research outcome. Third, despite the combined use of clinical symptoms, radiographic findings, and mNGS test for PCP diagnosis, the possibility of including patients with *P. jirovecii* colonization cannot be completely eliminated. Finally, the study participants were all enrolled from ICU, so the patients' condition was critical and severely ill. Therefore, our research outcome may be inapplicable for mild patients. Further studies are still needed among patients with PCP.

In conclusion, mNGS is a very sensitive diagnostic tool for identifying *P. jirovecii* inpatients who are non-HIV immunocompromised. PCP in patients who are non-HIV-infected is associated with a high rate of multiple infections and severe condition. Mixed infection and NLR were the independent risk factors of poor prognosis.

## Data Availability Statement

The data presented in the study are deposited in the NCBI repository. Accession numbers, SRA: SRP356529, Bioproject: PRJNA782857.

## Ethics Statement

The studies involving human participants were reviewed and approved by the Ethical Committee of The First Affiliated Hospital of Zhengzhou University. The patients/participants provided their written informed consent to participate in this study.

## Author Contributions

JD and JG participated in the research design, data analysis, and writing of the paper. QL and MS participated in data analysis and revising of the paper. YL and YT participated in the improving and revising of the paper. LX provided substantial advice in designing the study and assisting in the division of labor, writing, and revising the paper. All authors contributed to the article and approved the submitted version.

## Funding

This study was supported by the National Natural Science Foundation of China (82074212) and Key Medical Technologies R&D Program of Henan Province (SB201901036).

## Conflict of Interest

The authors declare that the research was conducted in the absence of any commercial or financial relationships that could be construed as a potential conflict of interest.

## Publisher's Note

All claims expressed in this article are solely those of the authors and do not necessarily represent those of their affiliated organizations, or those of the publisher, the editors and the reviewers. Any product that may be evaluated in this article, or claim that may be made by its manufacturer, is not guaranteed or endorsed by the publisher.
